# Prognostic nutritional index combined with NLR to construct a survival prediction model and decision analysis of patients with muscle‐invasive bladder cancer after surgery

**DOI:** 10.1002/cam4.6088

**Published:** 2023-05-18

**Authors:** Jincheng Wang, Hongjin Shi, Zhinan Fan, Jiaxin Yang, Yanghuang Zheng, Dan Zeng, Jinsong Zhang, Bing Hai

**Affiliations:** ^1^ Department of Urology, The Second Affiliated Hospital Kunming Medical University Kunming China; ^2^ Department of Urology People's Hospital of Luliang County Qujin China; ^3^ Department of Respiratory Medicine, The Second Affiliated Hospital Kunming Medical University Kunming China

**Keywords:** muscle‐invasive bladder cancer, neutrophil–lymphocyte ratio, nomogram, overall survival, prognostic nutritional index

## Abstract

**Objectives:**

To build a nomogram prediction model, assess its predictive ability, and perform a survival decision analysis on patients with muscle‐invasive bladder cancer (MIBC) to study risk factors affecting overall survival (OS).

**Methods:**

A retrospective analysis was performed on the clinical information of 262 patients with MIBC who underwent radical cystectomy (RC) at the Urology Department of the Second Affiliated Hospital of Kunming Medical University between July 2015 and August 2021. The final model variables that were included were chosen using single‐factor stepwise Cox regression, optimal subset regression, and LASSO regression + cross‐validation with the minimum AIC value. The next step was to do a multivariate Cox regression analysis. The establishment of a nomogram model by fitting and the screening out of independent risk factors impacting the survival of patients with MIBC having radical resection. Receiver Activity Characteristic curves, C‐index, and a calibration plot evaluated the prediction accuracy, validity, and clinical benefit of the model. The 1‐, 3‐, and 5‐year survival rates were then computed for each risk factor using a Kaplan–Meier survival analysis.

**Results:**

262 eligible patients in total were enrolled. With a median follow‐up of 32 months, the follow‐up period ranged from 2 to 83 months. 171 cases (65.27%) survived while 91 cases (34.73%) perished. Age (HR = 1.06 [1.04; 1.08], *p* = 0.001), preoperative hydronephrosis (HR = 0.69 [0.46, 1.05], *p* = 0.087), T stage (HR = 2.06 [1.09, 3.93], *p* = 0.027), lymphovascular invasion (LVI, HR = 1.73 [1.12, 2.67], *p* = 0.013), prognostic nutritional index (PNI, HR = 1.70 [1.09, 2.63], *p* = 0.018), and neutrophil‐to‐lymphocyte ratio (NLR, HR = 0.52 [0.29, 0.93)], *p* = 0.026) were independent risk factor for the survival of bladder cancer patients. Create a nomogram based on the aforementioned findings, and then draw the 1‐year, 3‐year, and 5‐year OS receiver operating characteristic curves by the nomogram. The AUC values were 0.811 (95% CI [0.752, 0.869]), 0.814 (95% CI [0.755, 0.873]), and 0.787 (95% CI [0.708, 0.865]), respectively, and the calibration plot matched the predicted value well. The 1‐year, 3‐year, and 5‐year decision curve analyses were higher than the ALL line and None line at threshold values of >5%, 5%–70%, and 20%–70% indicating that the model has good clinical applicability. The calibration plot for the Bootstrap 1000‐time resampled validation model was similar to the actual value. Patients with preoperative combination hydronephrosis, higher T‐stage, combined LVI, low PNI, and high NLR had worse survival, according to Kaplan–Meier survival analysis for each variable.

**Conclusions:**

This study might conclude that PNI and NLR were separate risk factors that affect a patient's OS after RC for MIBC. The prognosis of bladder cancer may be predicted by PNI and NLR, but additional confirmation in randomized controlled trials is required.

## 1 INTRODUCTION

The most frequent malignancy of the urinary system and the tenth most common malignancy overall is bladder cancer. It occurs more frequently and in younger people.^1^ Non‐muscle invasive bladder cancers (NMIBC), which affect about 70% of patients, are the most common type of bladder cancer. Radical cystectomy (RC), which includes pelvic lymph node dissection (PLND) and urine diversion, is the gold standard treatment for patients with numerous and recurrent NMIBC, coupled carcinoma in situ, high‐grade histology, and muscle‐invasive bladder cancer (MIBC). Patients who had RC had a 66% 5‐year survival rate.^2^ However, recovery from surgery depends on factors like age, gender, TNM stage, histological grade, preoperative Hydronephrosis,^3^, ^4^, ^5^, ^6^ combined lymphovascular invasion (LVI),^7^, ^8^, ^9^ preoperative nutritional state, and immunological status.^10^

Reduced post‐operative survival rates are a result of radical cystectomy's high level of invasiveness, higher T‐stage bladder cancer, and patients' suboptimal nutritional and immunological conditions before surgery.^11^, ^12^, ^13^ Therefore, the prevalence and progression of malignant tumors are significantly influenced by the preoperative nutritional state, systemic inflammatory response, and immunological function.^11^, ^12^, ^13^ A systematic review conducted by Ornaghi et al.^14^ revealed that preoperative hypoproteinemia was significantly associated with an increased incidence of postoperative complications in patients undergoing RC. Furthermore, individuals with preoperative hypoproteinemia exhibited poorer 3‐year overall survival (OS) rates. Multiple studies^15^, ^16^, ^17^ have found that preoperative hypoalbuminemia is significantly associated with an increased risk of cancer‐specific and total mortality as well as 30‐day morbidity. The tumor microenvironment triggers the release of cytokines, which in turn alters the systemic inflammatory response.^18^ Pretreatment measurements of systemic inflammatory responses such as lymphocytes, neutrophils, monocytes, platelets, and C‐reactive protein are inexpensive and widely available routine biomarkers. For example, the prognostic nutritional index (PNI) and neutrophil‐to‐lymphocyte ratio (NLR) have been used as scores and ratios in many studies.

PNI can be used to assess a patient's health status, surgical risk, and postoperative complications in the early stages of their illness, based on the serum albumin level and the total number of lymphocytes. It reflects the nutritional and immune status of patients with malignant tumors. Numerous studies have demonstrated that PNI is a reliable prognostic indicator for a variety of tumor types and is an independent risk factor for predicting malignant tumors.^19^, ^20^, ^21^, ^22^ There have been a lot of studies on urological tumor prediction using it in recent years,^23^, ^24^, ^25^, ^26^, ^27^, ^28^ but there have been fewer studies on MIBC. NLR, a reliable measure of the systemic inflammatory response, has also been demonstrated to be linked with the prognosis of several solid tumors, including bladder, lung, and stomach cancer.^29^, ^30^ However, there is a lack of literature on the combination of PNI and NLR in predicting the OS and complications of bladder cancer.

The Nomogram, a straightforward graphical representation of a statistical prediction model, is frequently used to forecast cancer prognosis by estimating the likelihood of a clinical event. The Nomogram is recommended as an alternative or perhaps as a new standard because it outperforms the conventional TNM staging system for many malignancies. Make customized predictions using the Nomograms to classify and identify patients for clinical trial participation. Combined with the above research background, for patients with MIBC who underwent RC, a survival prediction model and decision analysis were created in this study using PNI and NLR.

## 2 MATERIALS AND METHODS

### 2.1 Study population

The clinical data of 262 patients with MIBC who underwent RC from July 2015 to August 2021 in the Department of Urology of Second Affiliated Hospital of Kunming Medical University were retrospectively analyzed. Inclusion criteria: (I) pathologically confirmed MIBC; (II) RC performed; (III) complete routine blood and blood biochemistry data 1 week before surgery; (IV) immunohistochemical indices such as Ki 67, P53 and P63 available; (V) regular post‐operative follow‐up. Exclusion criteria: (I) concurrent other tumors; (II) concurrent other systemic significant diseases such as inflammatory diseases, liver diseases, auto‐immune diseases, hematological diseases, and cardiovascular diseases; (III) pre‐ and post‐operative co‐infectious diseases; (IV) severe complications in the perioperative period; (V) patients receiving pre‐operative neoadjuvant therapy; (VI) patients with no follow‐up records or missed follow‐ups. All patients had no preoperative neoadjuvant therapy, no other major systemic diseases in combination, and no severe complications in the perioperative period. The Ethics Committee of the Second Affiliated Hospital of Kunming Medical University gave its clearance to this investigation (approval number: audit‐pj‐2015‐102). The study was done by the Declaration of Helsinki, and each participant provided their informed permission.

### 2.2 Research methods

The blood routine and blood biochemical data of all patients within 1 week before the operation was calculated according to the following formula: NLR = neutrophils/lymphocytes (×10^9^/L); PNI = serum albumin (g/L) +5 × peripheral blood lymphocytes cells (×10^9^/L). Kaplan–Meier survival analysis based on a log‐rank test determined the optimal cut‐off values of PNI, NLR, and Ki‐67 and divided them into high and low groups.

### 2.3 Follow up

Follow‐up is recommended every 3–6 months for the first 1–2 years following surgery, then every 6–12 months. Regular blood tests, biochemical assessments, chest X‐rays, abdominal ultrasounds, and CT scans are all part of follow‐up care. The period of survival covered the period from the operation date to the date of death, loss of follow‐up, or loss of follow‐up in August 2022.

### 2.4 Surgical methods

The bladder and its surrounding fatty tissue, the distal ureter, the pelvic lymph nodes, the prostate, and the seminal vesicles are removed in male patients during surgery using the robotic laparoscopic RC, or laparoscopic RC, or open RC + urinary diversion techniques. The uterus, both bilateral adnexa, and a portion of the anterior vaginal wall is all resected in the case of the female patient. The cutaneous ureterostomy, ileal conduit (Bricker's bladder), and orthotopic neobladder are all examples of urinary diversions.

### 2.5 Statistical methods

For statistical analysis and the creation of statistical graphics, R4.2.1 software was utilized. Count data were presented as percentages and tested using either Fisher's exact probability test or the *x* ^2^ test. The data were expressed as $\bar{x}\pm \bar{s}$ and the independent sample *t*‐test was run if the measurement data fit the normal distribution and homogeneity of variance. The data were reported as median [IQR] and the nonparametric rank‐sum test (Wilcoxon test) was run if the data did not fit a normal distribution and/or the variance was not uniform. Univariate Cox regression analysis, optimal subset regression, LASSO regression, and cross‐validation were used to find the best set of variables. The multivariate COX analysis included the factors that were screened by the three techniques. The final filtered variables of the three methods were determined using the backward stepwise regression method, and the model with the smallest Akaike Information Criterion (AIC) value was constructed. Among the three comparison methods, the model with the smallest AIC value was used to construct the Nomogram. Nomograms were created to predict 1‐, 3‐, and 5‐year OS based on multivariate models. The Nomogram used a graphical depiction to connect several prognostic risk variables of patients to the likelihood of post‐operative OS in MIBC patients. The area under the receiver operating characteristic (ROC) curve, also known as the Concordance index (C‐index), which measured the Nomogram's discrimination, was almost identical to the area under the ROC curve. Using a calibrated plot, the Nomogram's calibration was intuitively assessed. When the model's anticipated value and the patient's risk match, the 45° line denoted a flawless calibration. Under‐prediction or over‐prediction, respectively, was indicated by deviations above or below the 45° line. The clinical value of the Nomogram was confirmed using the decision curve analysis (DCA). A curve that was above the None line and the All line denoted a net advantage and suggested that the model was more clinically applicable. The cutoff for this study's statistical significance was *p* < 0.05. (Download version R4.2.1 from Tsinghua University and the TUNA Team in China at mirrors.tuna.tsinghua.edu.cn/CRAN/).

## 3 RESULTS

### 3.1 Baseline characteristics of patients

**FIGURE 1:**
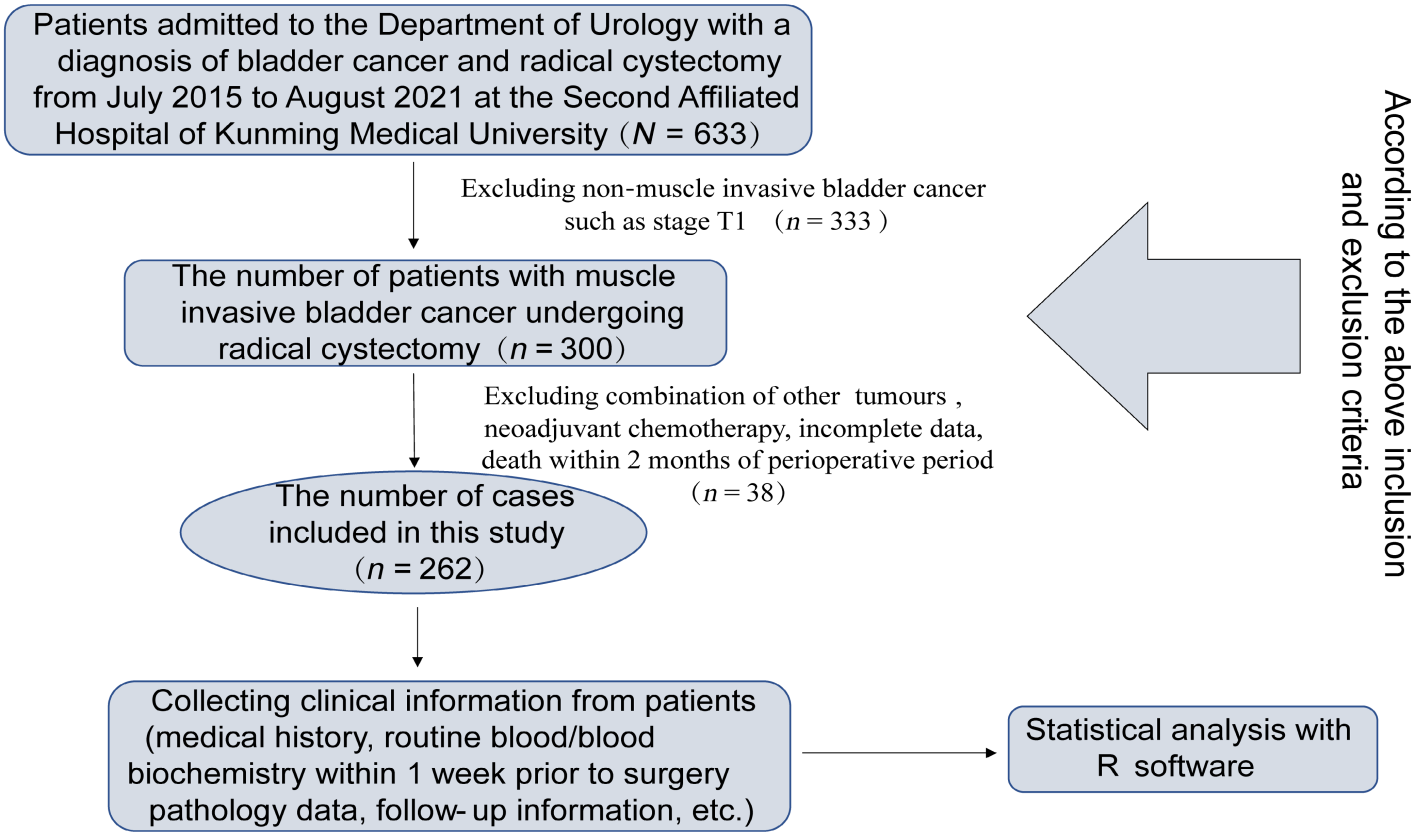
The screening variable process and technology roadmap for this study.

**FIGURE 2:**
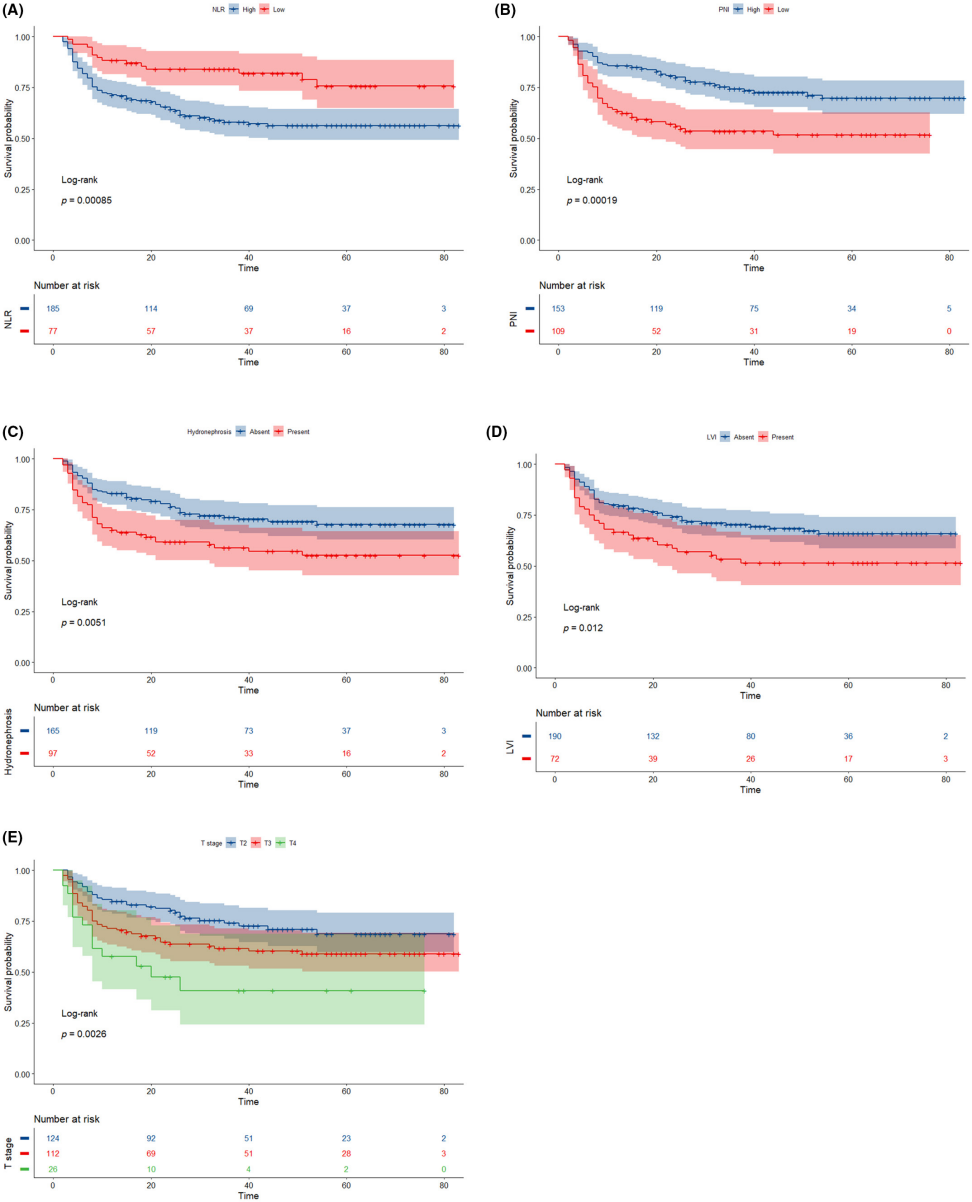
Kaplan–Meier curves demonstrating cause‐specific survival based on the status of NLR (A), PNI (B), Hydronephrosis (C), LVI (D), and T stage (E) (log‐rank test *p*‐value <0.05). LVI, lymphovascular invasion; NLR, neutrophil‐to‐lymphocyte ratio; PNI, prognostic nutritional index.

Based on the inclusion and exclusion criteria, 262 cases were finally included (Figure 1). In this study, the baseline clinical data of 262 patients were followed up for 2–83 months, with a median follow‐up time of 32 months (Table 1); 91 cases (34.73%) died and 171 cases (65.27%) survived. The optimal cut‐off values of PNI, NLR, and Ki‐67 determined by Kaplan–Meier survival analysis were 47.70%, 1.94, and 32.0%, respectively, divided into high and low groups. And the results showed 153 patients with high PNI (58.4%), 109 patients with low PNI (41.6%), 185 patients with high NLR (70.6%), 185 patients with low NLR patients 77 (29.4%), low Ki‐67‐expressing patients 80 (32.1%) and high Ki‐67‐expressing patients 169 (67.9%), and Kaplan–Meier survival analysis for each variable suggested poorer survival in patients with pre‐operative combined Hydronephrosis, higher T‐stage, combined LVI, low PNI, and high NLR (Figure 2). About the surgical margin, there were 8 (3.05%) positive urethral surgical margins (PSM) and 28 (10.69%) positive ureteral surgical margins. However, Main complications were postoperative intestinal obstruction (7.6%), urinary tract infection (20.23%), urinary incontinence (3.05%), dysuria (3.05%), post‐ureteral stenosis (1.91%), pulmonary embolism (0.38%), lower limb venous thrombosis (0.76%), and intestinal fistula (0.76%). After statistical analysis, it was found that PNI and NLR had no statistical significance in predicting the above complications (*p* > 0.05). Urothelial carcinomas (UC) with 63 (24%) squamous differentiation, 11 (4.2%) glandular differentiation, 3 (1.15%) micropapillary, 1 (0.38%) lymphoepithelioma‐like, 1 (0.38%) microcystic, 1 (0.38%) clear‐cell, 1 (0.38%) neuroendocrine variant, 1 (0.38%) small‐cell cases. 1 case (0.38%) each of simple intestinal adenocarcinoma, plasmacytoid (0.38%), and 2 cases (0.76%) each of simple lymphoepithelioma (Table 1). In addition, we also collected data on the patients' adjuvant therapy. 98 patients (T3, T4, or N+) were given 4 or 5 cycles of adjuvant therapy with GC regimen or GC chemotherapy combined with immunotherapy after surgery. The chemotherapy regimen used gemcitabine/cisplatin (GC) and was administered based on CSCO guidelines (gemcitabine, 1000 mg/m^2^ d1 and d8; cisplatin 70 mg/m^2^ d2, 21‐day cycle for 4 or 5 cycles).

**TABLE 1:** Clinical baseline information of 262 patients and analysis of factors influencing post‐operative OS in patients with MIBC.

Variables	*n*%/M[P_25_, P_75_]	Groups, *n*%/M[P_25_, P_75_]		*p* value
	*N* = 262	Survival group (*n* = 171, 65.27%)	Death group (*n* = 91, 34.73%)	
Gender				0.284
Male	236 (90.1)	157 (91.8)	79 (86.8)	
Women	26 (9.9)	14 (8.2)	12 (13.2)	
Age	66.00 [56.00, 73.00]	63.00 [33.00, 87.00]	71.00 [48.00, 86.00]	<0.001*
History of diabetes				0.096
Yes	18 (6.9)	8 (4.7)	10 (11.0)	
No	244 (93.1)	163 (95.3)	81 (89.0)	
History of hypertension				0.043*
Yes	50 (19.1)	26 (15.2)	24 (26.4)	
No	212 (80.9)	145 (84.8)	67 (73.6)	
History of smoking				0.937
Yes	129 (49.2)	85 (49.7)	44 (48.4)	
No	133 (50.8)	86 (50.3)	47 (51.6)	
Pre‐operative hydronephrosis				0.018*
Yes	97 (37.0)	54 (31.6)	43 (47.3)	
No	165 (63.0)	117 (68.4)	48 (52.7)	
Maximum tumor diameter				0.069
≤1 cm	47 (17.9)	30 (17.5)	17 (18.7)	
1–3 cm	65 (24.8)	50 (29.2)	15 (16.5)	
≥3 cm	150 (57.3)	91 (53.2)	59 (64.8)	
Number of tumors				0.071
Multiple	89 (34.0)	51 (29.8)	38 (41.8)	
Single	173 (66.0)	120 (70.2)	53 (58.2)	
Histological grading				0.459
HG	154 (58.8)	96 (56.1)	58 (63.7)	
LG	14 (5.3)	12 (7.0)	2 (2.2)	
HG + LG	12 (4.6)	9 (5.3)	3 (3.3)	
HG + other	77 (29.4)	51 (29.8)	26 (28.6)	
LG + other	1 (0.4)	1 (0.6)	0 (0.0)	
Other	4 (1.5)	2 (1.2)	2 (2.2)	
T stage				0.012*
T2	124 (47.3)	91 (53.2)	33 (36.3)	
T3	112 (42.7)	68 (39.8)	44 (48.4)	
T4	26 (9.9)	12 (7.0)	14 (15.4)	
Presence of lymph node metastases				0.181
Absent	240 (91.6)	160 (93.6)	80 (87.9)	
Present	22 (8.4)	11 (6.4)	11 (12.1)	
M stage				0.347
M0	261 (99.6)	171 (100.0)	90 (98.9)	
M1a	1 (0.4)	0 (0.0)	1 (1.1)	
Lymphovascular invasion				0.029*
Absent	190 (72.5)	132 (77.2)	58 (63.7)	
Present	72 (27.5)	39 (22.8)	33 (36.3)	
P63				0.934
−	37 (15.5)	24 (15.1)	13 (16.5)	
+	201 (84.5)	135 (84.9)	66 (83.5)	
P53				0.901
−	66 (30.8)	45 (31.5)	21 (29.6)	
+	148 (69.2)	98 (68.5)	50 (70.4)	
Ki‐67	40.0% [30.0%, 60.0%]	40.0% [5.0%, 80.0%]	50.0% [5.0%, 90.0%]	0.301
PNI	48.45 [43.59, 51.74]	49.25 [32.30, 66.10]	47.15 [16.37, 61.40]	0.004*
NLR	2.47 [1.79, 3.57]	2.35 [0.76, 15.20]	2.64 [0.90, 21.22]	0.008*
PNI groups				0.002*
High	153 (58.4)	112 (65.5)	41 (45.1)	
Low	109 (41.6)	59 (34.5)	50 (54.9)	
NLR groups				0.001*
High	185 (70.6)	109 (63.7)	76 (83.5)	
Low	77 (29.4)	62 (36.3)	15 (16.5)	
Ki‐67 groups				0.076
High	169 (67.9)	106 (63.9)	63 (75.9)	
Low	80 (32.1)	60 (36.1)	20 (24.1)	
Surgical approach				1.000
Laparoscopic surgery	226 (86.26)	148 (86.55)	78 (85.71)	
Open surgery	36 (13.74)	23 (13.45)	13 (14.29)	
Status				
Survival	171 (65.3)			
Death	91 (34.7)			
Follow‐up time	32.00 [12.00, 56.00]			

*Note*: *p < 0.05, the difference is statistically significant in bold.

Abbreviations: HG, high‐grade urothelial carcinoma; LG, low‐grade urothelial carcinoma.

### 3.2 Factors affecting the overall survival of MIBC Patients after RC

Among the 262 patients, 91 (34.73%) died, and 171 (65.27%) survived. Each potential risk factor was analyzed separately to see whether it affected the OS of the patients. Statistical results showed (Table 1) that there was no statistically significant difference between patients' gender, history of diabetes, maximum tumor diameter, Presence of multiple tumors, histological grading, lymph node metastasis, M‐stage, P63, P53, Ki‐67 and its subgroups and surgical approach from OS (*p* > 0.05), while age history of hypertension, presence of pre‐operative Hydronephrosis, T‐stage, Presence of LVI, PNI and NLR and their subgroups were statistically significantly different from OS (*p* < 0.05).

**FIGURE 3:**
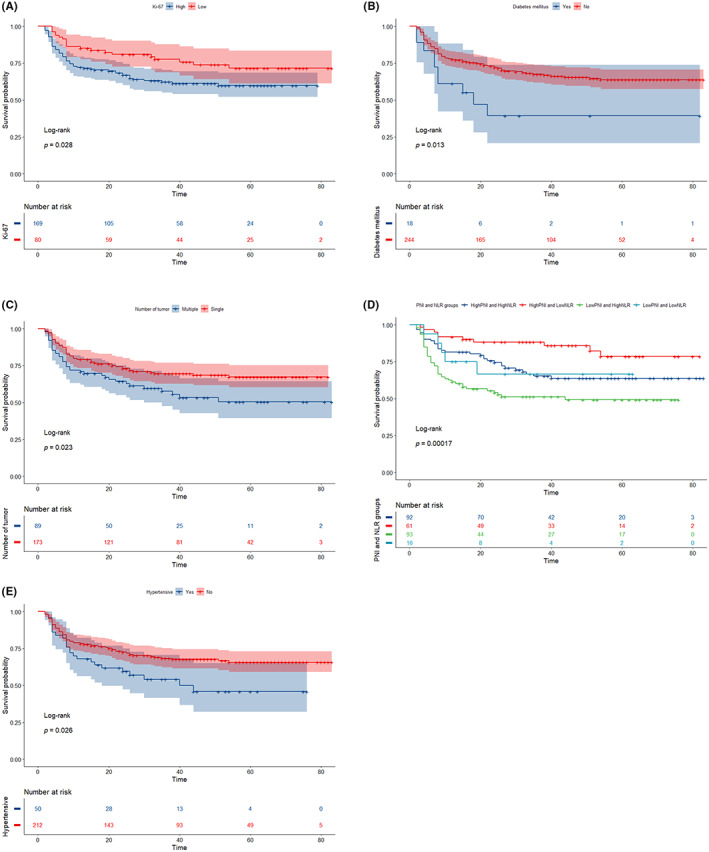
Kaplan–Meier curves demonstrating cause‐specific survival based on the status of Ki‐67 (A), diabetes mellitus (B), number of tumors (C), PNI and NLR (D), and hypertensive (E) (log‐rank test *p*‐value <0.05). NLR, neutrophil‐to‐lymphocyte ratio; PNI, prognostic nutritional index.

The aforementioned statistically significant risk factors were subjected to a univariate Cox regression analysis (Table 2). Age, history of hypertension, preoperative hydronephrosis, the T stage, LVI, PNI, and NLR were found to be risk factors for OS in MIBC patients following RC (*p* < 0.05). The groups for PNI and NLR were high and low, respectively. The two variables were then reorganized into four groups: high PNI low NLR, high PNI high NLR, low PNI low NLR, and low PNI high NLR. According to Kaplan‐log‐rank Meier's test for survival analysis (Figure 3D), the groups with high PNI and low NLR had the best survival rates, while the groups with low PNI and high NLR had the lowest. Between the high PNI and high NLR group and the low PNI and low NLR group, there was no discernible difference (*p* < 0.05).

**TABLE 2:** Analysis of factors influencing the prognosis of patients with MIBC after radical cystectomy.

Variables	Number (%)	Single‐factor COX regression		Multi‐factor COX regression	
		HR [95% CI]	*p* value	HR [95% CI]	*p* value
Age	64.45 (±11.09)	1.06 [1.04, 1.09]	<0.001	1.06 [1.04, 1.08]	<0.001
History of hypertension					
Yes	50 (19.1)				
No	212 (80.9)	0.59 [0.37, 0.94]	0.028	0.99 [0.60, 1.61]	0.957
Pre‐operative hydronephrosis					
Yes	97 (37.0)				
No	165 (63.0)	0.56 [0.37, 0.84]	0.005	0.69 [0.46, 1.05]	0.087
T stage					
T2	124 (47.3)				
T3	112 (42.7)	1.61 [1.02, 2.53]	0.039	1.49 [0.95, 2.35]	0.085
T4	26 (9.9)	2.87 [1.53, 5.38]	0.001	2.06 [1.09, 3.93]	0.027
Lymphovascular invasion					
Absent	190 (72.5)				
Present	72 (27.5)	1.72 [1.12, 2.63]	0.013	1.73 [1.12, 2.67]	0.013
PNI groups					
High	153 (58.4)				
Low	109 (41.6)	2.17 [1.43, 3.28]	<0.001	1.70 [1.09, 2.63]	0.018
NLR groups					
High	185 (70.6)				
Low	77 (29.4)	0.4 [0.23, 0.7]	0.001	0.52 [0.29, 0.93]	0.026

*Note*: *p* < 0.05, the difference is statistically significant.

The risk factors obtained from the above single‐factor Cox regression analysis were subjected to multi‐factor Cox regression analysis (Table 2). Stepwise backward regression was used to determine the best combination of variables for constructing the model with the minimum AIC value. At that age, pre‐operative hydronephrosis, T‐stage, Presence of LVI, PNI, and NLR were independent risk factors for OS after RC in patients with MIBC (*p* < 0.05). Among them, the risk factors discovered by the aforementioned univariate Cox regression analysis were submitted to multivariate Cox regression analysis (Table 2), and the optimal variable combination with the minimum AIC value was found by a stepwise backward regression approach to create a model. When patients with MIBC underwent RC at age, pre‐operative hydronephrosis, T‐stage, the presence of LVI, PNI, and NLR were independent risk factors for OS following the procedure (*p* < 0.05). The presence of pre‐operative hydronephrosis was *p* > 0.05 among them. Even yet, using techniques like cross‐validation and optimal subset regression, the presence of preoperative hydronephrosis was identified as one of the most effective sets of factors. Preoperative hydronephrosis was proven to be a separate risk factor for survival following RC for bladder cancer in earlier research.^3^, ^4^, ^5^ Preoperative hydronephrosis was thus incorporated into the model in this investigation. According to the Kaplan–Meier survival analysis with the Log‐rank test for PNI, NLR, LIV, Ki‐67, T‐stage, and pre‐operative combined hydronephrosis, survival was worse for low PNI, high NLR, combined LVI, high Ki‐67 expression, higher T‐stage, pre‐operative combined hydronephrosis with multiple tumors, and combined with hypertensive diabetes (Figures 2 and 3).

### 3.3 Prediction model construction, evaluation, and validation

#### 3.3.1 The Nomograms predict 1‐, 3‐, and 5‐year OS in MIBC patients after radical cystectomy

**FIGURE 4:**
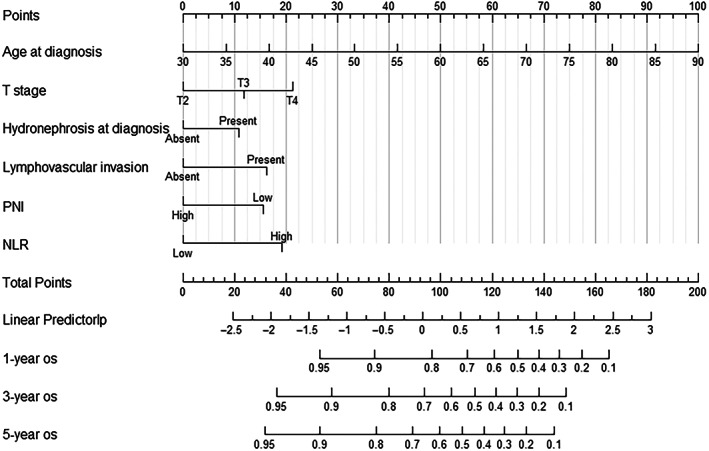
The Nomogram influencing bladder cancer survival. NLR, neutrophil‐to‐lymphocyte ratio; PNI, prognostic nutritional index.

According to the results of multivariate Cox analysis, independent risk factors such as age, preoperative hydronephrosis, T stage, lymphatic vessel invasion, PNI, and NLR were fitted to construct the Nomogram of the 1‐year, 3‐year, and 5‐year OS columns after RC in MIBC patients (Figure 4).

#### 3.3.2 Evaluation and validation of the model

This study used internal validation to assess and validate each of the three aspects of the model: discrimination, calibration, and clinical benefit.

##### Validation of the model for differentiation or accuracy assessment

**FIGURE 5:**
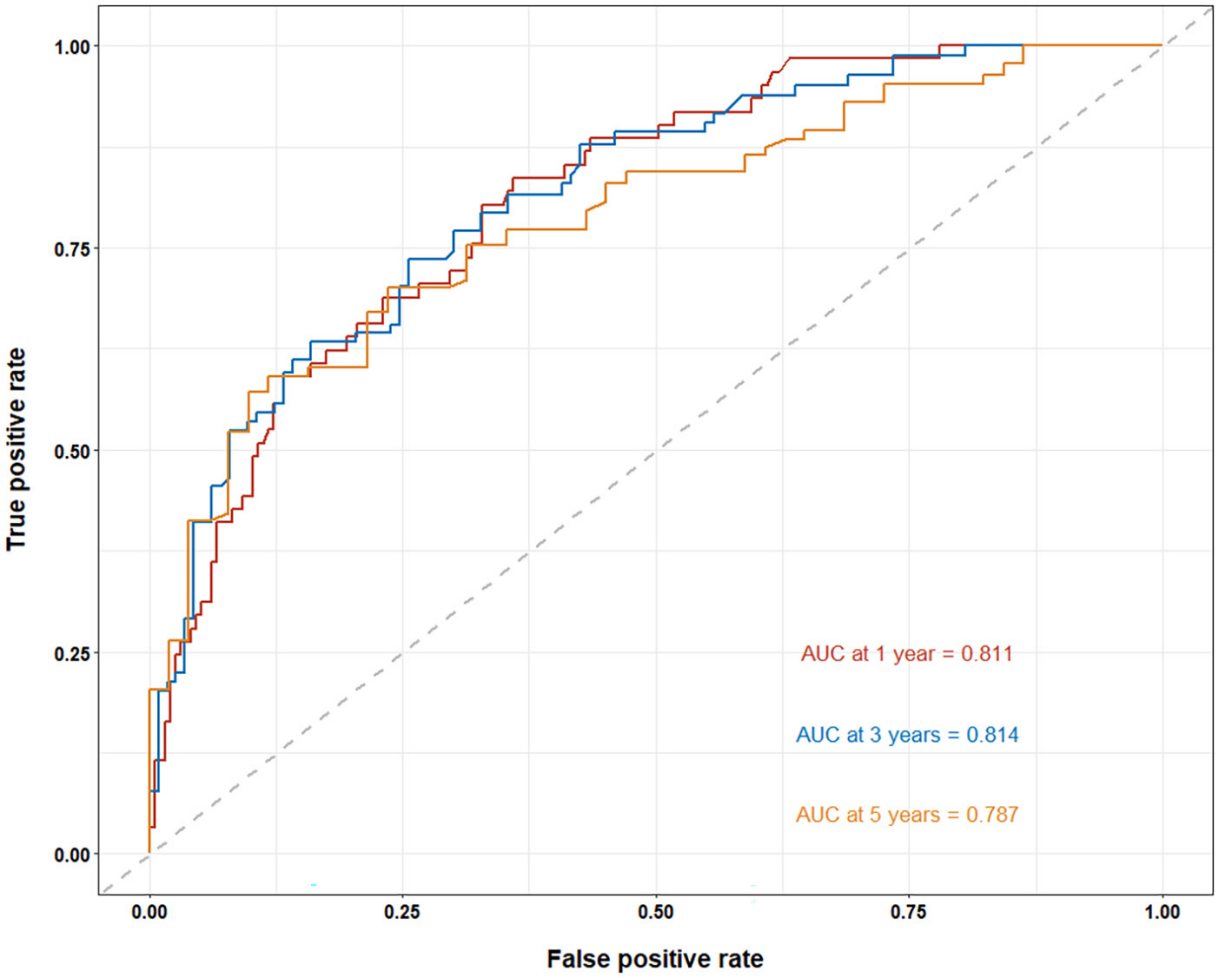
Receiver operating characteristic of the Nomogram.

**FIGURE 6:**
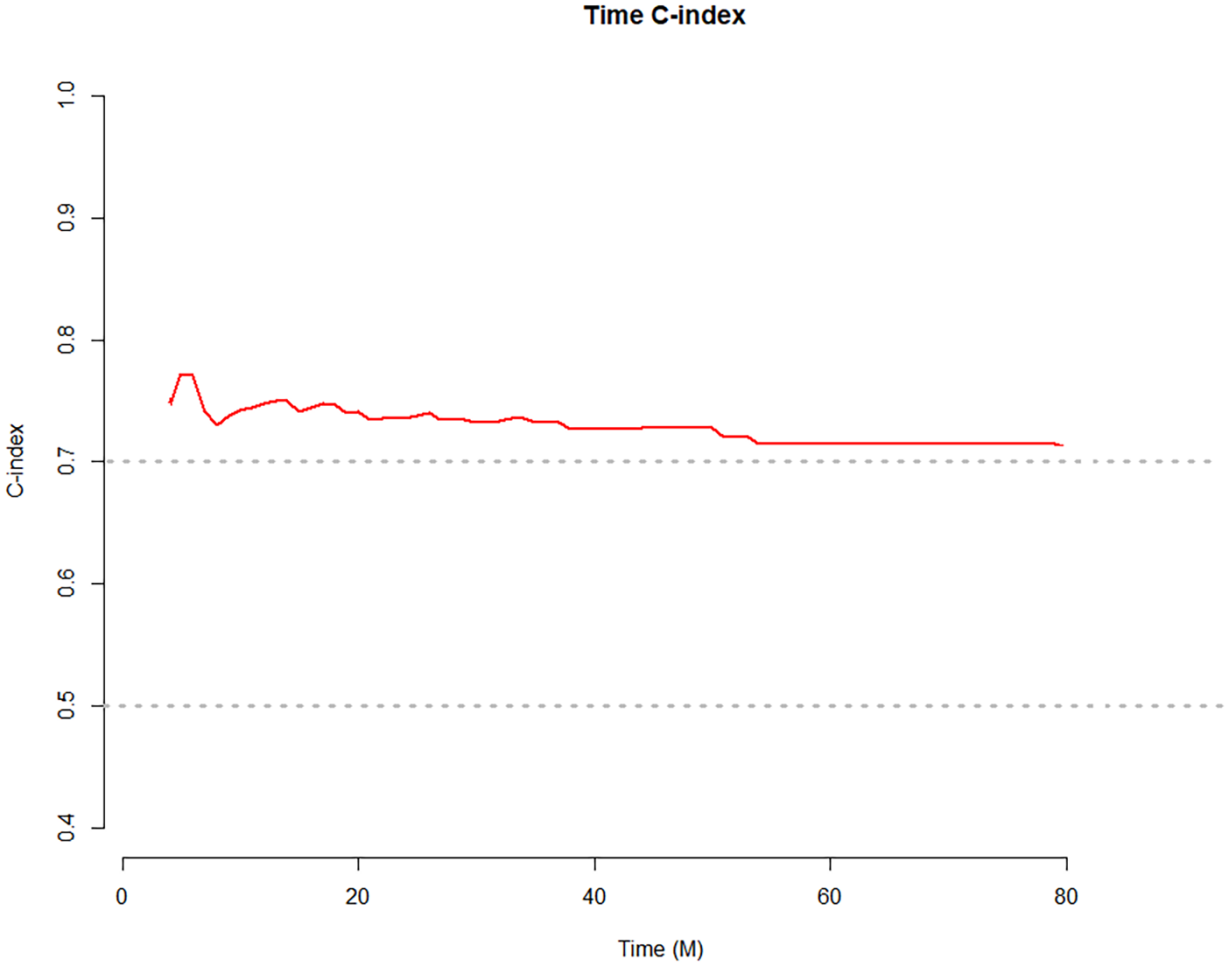
Time C‐index of the Nomogram.

The ability to predict 1‐year OS, 3‐year OS, and 5‐year OS was evaluated using the C‐index and the area under the ROC curve (Figure 5). For predicting 1‐year OS, 3‐year OS, and 5‐year OS, the AUC values, 95% CI, sensitivity (Se), specificity (Sp), and Youden index (YI) were good (Table 3). The 95% CI for the nomogram's C‐index was between 0.710 and 0.798. The Nomogram had a high predictive value, as indicated by the C‐index, which was greater than 0.7. The C‐index changed over the course of the follow‐up period (Figure 6), but it was consistently higher than 0.7, showing that the Nomogram was reliable and stable.

**TABLE 3:** Comparison of AUC values for 1 to 5‐year OS for predictive models.

OS	AUC values	95% CI	Se	Sp	YI
1‐year OS	0.811	0.752–0.869	0.836	0.641	0.477
3‐year OS	0.814	0.755–0.873	0.735	0.744	0.479
5‐year OS	0.787	0.708–0.865	0.91	0.883	0.474

Abbreviations: 95% CI, 95% confidence interval; OS, overall survival; Se, sensitivity; Sp, specificity; YI, Youden index.

##### Validation and assessment of the model's calibration

**FIGURE 7:**
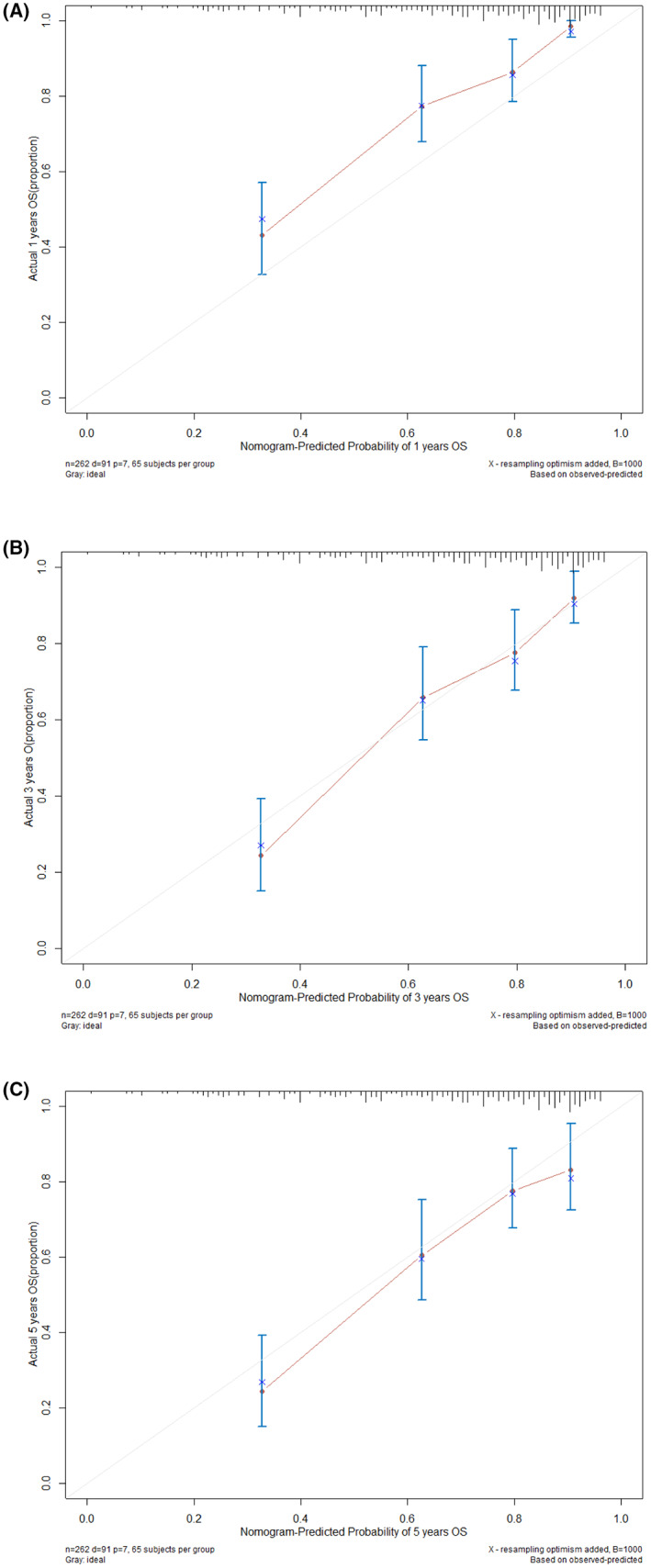
The Nomogram's calibrate plot of 1‐year OS (A), 3‐year OS (B), and 5‐year OS (C). OS, overall survival.

Utilize calibration plots and Bootstrap techniques to validate prediction models. For this study's internal validation procedure, the predictive model's precision was examined. The estimation of 1‐year, 3‐year, and 5‐year OS following RC was confirmed using 1000‐time resampling of raw data using the R soft. Calibration plots were also created (Figure 7A–C). The calibration curve for predicting patient survival at 1 year was above the 45° line, indicating a poor performance of the Nomogram in predicting survival at 1 year. The calibration plots performed well and accurately in predicting survival at 3 and 5 years, as seen by the calibration curve for floating around the 45° line.

##### Validation and assessment of the model for clinical decision

**FIGURE 8:**
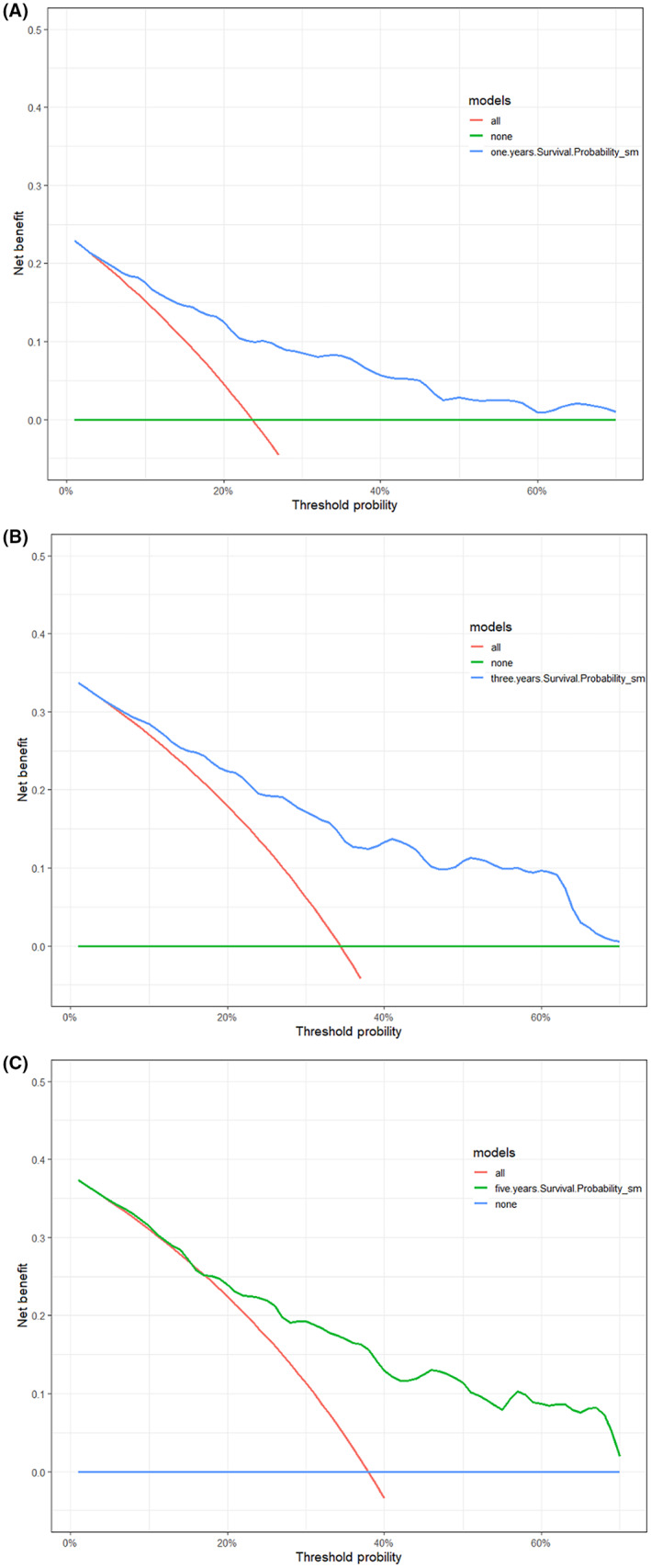
The Nomogram's decision curve analysis of 1‐year OS (A), 3‐year OS (B), and 5‐year OS (C). OS, overall survival.

**FIGURE 9:**
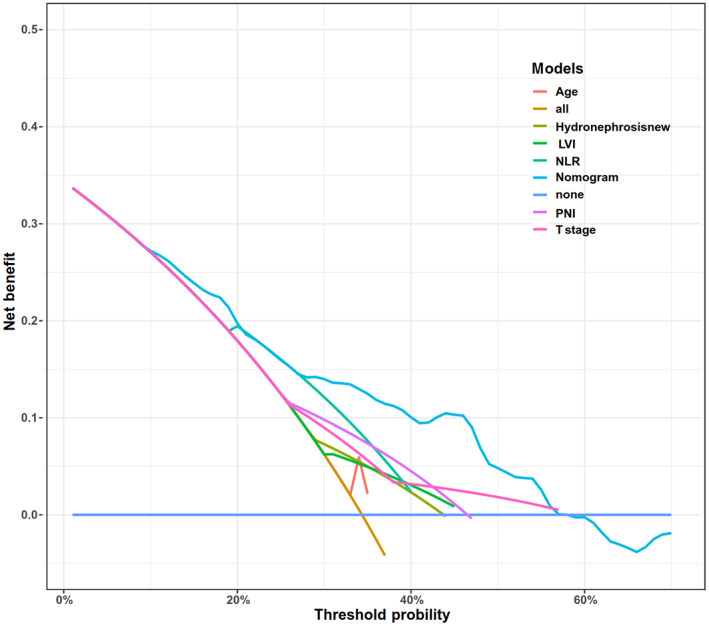
Decision curve analysis and comparison of variables in the Nomogram. LVI, lymphovascular invasion; NLR, neutrophil‐to‐lymphocyte ratio; PNI, prognostic nutritional index.

Decision curve analysis (DCA) was used to assess the extent of patient clinical benefit. 1‐year, 3‐year, and 5‐year decision curve analyses showed that the decision curves were always higher than the “None” line and “All” line, and the model has clinical applicability (Figure 8A–C). The DCA for each variable within the model was poorer than the model's DCA curve when compared to the model's net benefit. Still, both were above the None and All lines, indicating that the model had good clinical applicability (Figure 9).

## 4 DISCUSSION

The most prevalent malignant tumor of the urinary tract, bladder cancer has a high fatality rate, an increasing prevalence, and an aging population. MIBC is becoming more prevalent. The current gold standard still includes PLND and RC and neoadjuvant cisplatin‐based chemotherapy (NAC). The clinical questions, which reflect a variety of survival issues, include, however, OS and 1–5 year survival after radical cystectomy in patients with bladder cancer. This study developed and validated a nomogram model to predict OS following radical cystectomy using two prognostic variables, PNI and NLR. The Nomogram had a fantastic net clinical benefit, according to the DCA. As a result, the nomogram model from this study aids doctors in predicting OS and clinical choice following radical cystectomy.

PNI is a prognostic indicator first proposed by Buzby et al.^31^ for the combined assessment of serum albumin and lymphocytes. It is used to assess the risk of gastrointestinal surgery and nutritional and immune status. In addition, many studies have found that PNI is closely related to the prognosis of many tumors,^19^, ^20^, ^21^, ^22^ including bladder cancer, prostate cancer, and kidney cancer in urological tumors.^23^, ^24^, ^25^, ^26^, ^27^, ^28^ A dimension of PNI is serum albumin, and some clinical studies have found that low serum albumin levels were independently associated with bladder cancer recurrence and decreased postoperative OS.^14^, ^15^, ^16^ However, serum albumin is one of the most commonly used non‐invasive and reproducible clinical indicators for nutritional assessment. Malnutrition and hypoproteinemia were associated with adverse effects on postoperative complications and OS in bladder cancer.^14^, ^32^, ^33^ Currently, perioperative correction of malnutrition and improved immunity has improved OS for bladder cancer, but evidence from prospective studies is still needed to validate this.^32^, ^33^, ^34^ Another dimension of PNI is lymphocytes. The decrease in serum lymphocyte counts indirectly suggests the reduction of effector cells involved in tumor immunity, which weakens the body's anti‐tumor immune response. Low lymphocyte counts were associated with bladder cancer recurrence and poor OS.^34^, ^35^ Based on the above study background, the impact of serum albumin and peripheral blood lymphocytes on the prognosis of bladder cancer was analyzed comprehensively. This study found that low PNI (PNI < 47.6) was an independent risk factor for poor OS after radical cystectomy.

In many solid tumors, NLR has been used to predict survival and postoperative problems.^29^, ^30^ Neutrophils are one of the signs of NLR. As inflammatory response cells, neutrophils block the cytolytic activity of immune cells including lymphocytes, activated T lymphocytes, and N‐K cells, hence suppressing the immune system.^36^, ^37^ Other cells like macrophages and neutrophils secrete substances that stimulate the tumor microenvironment while inhibiting lymphocyte activity. These substances promote tumor growth, proliferation, invasion, and metastasis. These substances include vascular endothelial growth factor,^38^, ^39^ hepatocyte growth factor,^40^ IL‐6,^41^ IL‐8,^42^ matrix metalloproteinases,^43^ and elastase.^44^ High NLR indicated a neutrophil‐dependent inflammatory response that was augmented and a lymphocyte‐mediated anti‐tumor immune response that was diminished.^30^ These factors may promote tumor invasion and metastasis, tumor development, and a poor prognosis. After radical cystectomy, patients with high NLR had a worse prognosis and significantly shorter 5‐year OS than those with low NLR. NLR was identified as an independent risk factor for OS following radical cystectomy by multivariate Cox analysis.

LVI is crucial for spreading and engulfing bladder cancer cells. Positive LVI implied a substantial relationship between lymph node metastases, histological grade, and pathological stage.^7^, ^8^ Additionally, LVI occurred before or at the same time as lymph node metastases, but once this stage had been reached in a patient, LVI status lost its predictive significance for survival or recurrence. As a result, in node‐negative patients receiving radical cystectomy and PLND, LVI status was a significant predictor of survival and reproduction.^45^ Additionally, a combination of urine or serological indicators assisted in identifying bladder cancer patients who would benefit from neoadjuvant or adjuvant therapy.^45^ The results of other mate analyses also showed that LVI was a significant predictive factor for patients with bladder cancer who had a worse prognosis,^7^, ^8^, ^9^ with this association being particularly significant in bladder cancer that was both lymph node‐negative than lymph node‐positive.^46^ The existence of LVI was linked to lower OS in this sample of 262 patients (*p* = 0.012), and multivariate analysis revealed that LVI status was an independent prognostic factor for MIBC patients following RC.

Advanced tumor stage, positive lymph nodes, and positive surgical margins were all strongly correlated with preoperative hydronephrosis. Hydronephrosis was a risk factor on its own for disease‐free survival following RC and was connected to tumor‐specific survival.^3^, ^4^, ^5^, ^6^ According to the univariate Cox analysis conducted in this study, preoperative hydronephrosis was a risk factor for OS in MIBC patients. In multivariate Cox analysis, preoperative hydronephrosis did not independently increase the risk of OS in MIBC patients (*p* > 0.05). Based on earlier investigations, it was still nevertheless incorporated into the model. The accuracy and validity of the model were not affected by including preoperative hydronephrosis, and Kaplan–Meier survival analysis (Figure 2C) amply demonstrated that OS was poorer with preoperative hydronephrosis than without hydronephrosis.

A multi‐center, multi‐laboratory analysis in 1058 RC patients^47^ showed that P53 and Ki‐67 had no significant correlation with the prognosis of patients. This is consistent with the findings of the present study. In addition, Mertens et al.^47^ found that P53 and Ki‐67 expression levels were associated with adverse tumor characteristics of bladder cancer. However, in our hospital, only qualitative detection of P53, P63, KI‐67. Therefore, no further analysis was performed. In addition, some studies have shown that variant histology (VH) was significant for the prognosis of bladder cancer and that each VH has a different impact. Mori et al.^48^ concluded that micropapillary, plasmacytoid, and small‐cell VH had worse OS, which suggested an independent risk factor. Similarly, Claps et al.^49^ study found that clear‐cell, plasmacytoid, small‐cell, and sarcomatoid VH were associated with worse disease‐specific survival (DSS), and Moschini et al.^50^ study found that small‐cell VH was associated with decreased OS in RC patients. However, our study found that VH was a risk factor for OS but not an independent risk factor for bladder cancer and may be related to the small sample size. Many studies surgical margin and location also had an impact on the prognosis of bladder cancer, and our study sample suggested that PSM was a risk factor (*p* = 0.0131) but not an independent risk factor (*p* = 0.8618) for OS after RC. Both Claps et al.^51^ and Marcq et al.^52^ studies suggested that patients with PSM had a poor prognosis, and the prognosis is worse for multifocal PSM, urethral PSM, and soft‐tissue PSM.

The prediction model using age, presence of pre‐operative hydronephrosis, T‐stage, LVI, PNI, and NLR had a good C‐index and correlated well with the actual occurrence. The calibration plot also showed that the Nomogram could effectively predict OS after RC in patients with MIBC, which had good clinical application. The DCA results showed that the model had good clinical applicability.

The Nomogram can guide clinicians in making personalized clinical decisions and improve post‐operative follow‐up and timely identification of problems for appropriate management. In addition, the PNI and NLR used to construct the Nomogram were easy to obtain and low cost, which made the nomogram model clinically applicable. At the same time, there are some shortcomings in this study. First, this study was a retrospective study with a small sample, which prevented any bias in the follow‐up of patients. Second, the values of the Nomogram in this study were validated internally but not externally to validate the general applicability of the model further. Third, the endpoint of this study was death, and for various reasons, patients were not followed up for the time to recurrence. Tumor‐free survival and tumor‐specific survival were not described in this study. For another, this study did not include lymph node metastasis, which was different from previous studies. It may be related to the individual differences of the included patients and whether they underwent postoperative adjuvant chemotherapy. In addition, short follow‐up and low single‐center sample size were also limitations of this study. Finally, this study only analyzed factors other than surgery and did not investigate the prognostic impact of different urinary diversion methods.

## 5 CONCLUSION

In conclusion, this study can conclude that age, history of diabetes mellitus, history of hypertension, pre‐operative hydronephrosis, high T‐stage, presence of lymph node metastasis or regional extra‐lymph node metastasis, combined LVI, high Ki‐67 expression, low PNI, and high NLR were prognostic influencing factors affecting post‐operative RC in patients with MIBC; among them, age, pre‐operative hydronephrosis, high T‐stage, combined LVI, low PNI, and high NLR were independent risk factors affecting OS after RC in patients with MIBC. PNI and NLR may be predictive factors for predicting bladder cancer prognosis, but further validation in multicentre randomized controlled trials is needed.

## AUTHOR CONTRIBUTIONS

**Jincheng Wang:** Conceptualization (equal); data curation (equal); formal analysis (equal); investigation (equal); methodology (equal); resources (equal); software (equal); visualization (equal); writing – original draft (equal); writing – review and editing (equal). **Hongjin Shi:** Conceptualization (equal); data curation (equal); formal analysis (equal); investigation (equal); methodology (equal); resources (equal); software (equal); visualization (equal); writing – review and editing (equal). **Zhinan Fan:** Conceptualization (equal); data curation (equal); formal analysis (equal); methodology (equal); resources (equal); software (equal); visualization (equal); writing – review and editing (equal). **Jiaxin Yang:** Data curation (equal); investigation (equal); software (equal); visualization (equal). **Yanghuang Zheng:** Data curation (equal); investigation (equal); software (equal); visualization (equal). **Dan Zeng:** Data curation (equal); investigation (equal); software (equal); visualization (equal). **Jinsong Zhang:** Conceptualization (equal); data curation (equal); formal analysis (equal); funding acquisition (equal); methodology (equal); project administration (equal); software (equal); supervision (equal); writing – review and editing (equal). **Bing Hai:** Conceptualization (equal); data curation (equal); formal analysis (equal); methodology (equal); project administration (equal); resources (equal); supervision (equal); writing – review and editing (equal).

## FUNDING INFORMATION

This work was supported by the National Natural Science Foundation of China (Grant Nos. 82060464 and 81860452).

## Data Availability

N/A.

## References

[1] Sung H , Ferlay J , Siegel RL , et al. Global cancer statistics 2020: GLOBOCAN estimates of incidence and mortality worldwide for 36 cancers in 185 countries. CA Cancer J Clin. 2021;71(3):209‐249. 33538338 10.3322/caac.21660

[2] Quek ML , Stein JP , Daneshmand S , et al. A critical analysis of perioperative mortality from radical cystectomy. J Urol. 2006;175(3 Pt 1):886‐889. discussion 9‐90. 16469572 10.1016/S0022-5347(05)00421-0

[3] Bartsch GC , Kuefer R , Gschwend JE , de Petriconi R , Hautmann RE , Volkmer BG . Hydronephrosis as a prognostic marker in bladder cancer in a cystectomy‐only series. Eur Urol. 2007;51(3):690‐697. discussion 7‐8. 16904815 10.1016/j.eururo.2006.07.009

[4] Oh JJ , Byun SS , Jeong CW , Kwak C , Kim HH , Ku JH . Association between preoperative hydronephrosis and prognosis after radical cystectomy among patients with bladder cancer: a systemic review and meta‐analysis. Front Oncol. 2019;9:158. 30941309 10.3389/fonc.2019.00158 PMC6433994

[5] Zhu Z , Zhao J , Li Y , Pang C , Zhu Z , Zhang X . Prognostic value of preoperative hydronephrosis in patients with bladder cancer undergoing radical cystectomy: a meta‐analysis. PLoS ONE. 2019;14(9):e0222223. 31513614 10.1371/journal.pone.0222223 PMC6742405

[6] Scrimger RA , Murtha AD , Parliament MB , et al. Muscle‐invasive transitional cell carcinoma of the urinary bladder: a population‐based study of patterns of care and prognostic factors. Int J Radiat Oncol Biol Phys. 2001;51(1):23‐30. 11516847 10.1016/s0360-3016(01)01591-7

[7] Kim H , Kim M , Kwak C , Kim HH , Ku JH . Prognostic significance of lymphovascular invasion in radical cystectomy on patients with bladder cancer: a systematic review and meta‐analysis. PLoS ONE. 2014;9(2):e89259. 24586637 10.1371/journal.pone.0089259 PMC3931717

[8] Kim HS , Kim M , Jeong CW , Kwak C , Kim HH , Ku JH . Presence of lymphovascular invasion in urothelial bladder cancer specimens after transurethral resections correlates with risk of upstaging and survival: a systematic review and meta‐analysis. Urol Oncol. 2014;32(8):1191‐1199. 24954108 10.1016/j.urolonc.2014.05.008

[9] Tian YF , Zhou H , Yu G , et al. Prognostic significance of lymphovascular invasion in bladder cancer after surgical resection: a meta‐analysis. J Huazhong Univ Sci Technolog Med Sci. 2015;35(5):646‐655. 26489616 10.1007/s11596-015-1484-4

[10] Nakamura I , Shibata M , Gonda K , et al. Serum levels of vascular endothelial growth factor are increased and correlate with malnutrition, immunosuppression involving MDSCs and systemic inflammation in patients with cancer of the digestive system. Oncol Lett. 2013;5(5):1682‐1686. 23761834 10.3892/ol.2013.1231 PMC3678612

[11] Castillo‐Martinez L , Castro‐Eguiluz D , Copca‐Mendoza ET , et al. Nutritional assessment tools for the identification of malnutrition and nutritional risk associated with cancer treatment. Rev Invest Clin. 2018;70(3):121‐125. 29943772 10.24875/RIC.18002524

[12] Balkwill F , Mantovani A . Inflammation and cancer: back to Virchow? Lancet. 2001;357(9255):539‐545. 11229684 10.1016/S0140-6736(00)04046-0

[13] Lu H , Ouyang W , Huang C . Inflammation, a key event in cancer development. Mol Cancer Res. 2006;4(4):221‐233. 16603636 10.1158/1541-7786.MCR-05-0261

[14] Ornaghi PI , Afferi L , Antonelli A , et al. The impact of preoperative nutritional status on post‐surgical complication and mortality rates in patients undergoing radical cystectomy for bladder cancer: a systematic review of the literature. World J Urol. 2021;39(4):1045‐1081. 32519225 10.1007/s00345-020-03291-z

[15] Lambert JW , Ingham M , Gibbs BB , Given RW , Lance RS , Riggs SB . Using preoperative albumin levels as a surrogate marker for outcomes after radical cystectomy for bladder cancer. Urology. 2013;81(3):587‐592. 23352372 10.1016/j.urology.2012.10.055

[16] Djaladat H , Bruins HM , Miranda G , Cai J , Skinner EC , Daneshmand S . The association of preoperative serum albumin level and American Society of Anesthesiologists (ASA) score on early complications and survival of patients undergoing radical cystectomy for urothelial bladder cancer. BJU Int. 2014;113(6):887‐893. 23906037 10.1111/bju.12240

[17] Gupta D , Lis CG . Pretreatment serum albumin as a predictor of cancer survival: a systematic review of the epidemiological literature. Nutr J. 2010;9:69. 21176210 10.1186/1475-2891-9-69 PMC3019132

[18] Gakis G . The role of inflammation in bladder cancer. Adv Exp Med Biol. 2014;816:183‐196. 24818724 10.1007/978-3-0348-0837-8_8

[19] Okadome K , Baba Y , Yagi T , et al. Prognostic nutritional index, tumor‐infiltrating lymphocytes, and prognosis in patients with esophageal cancer. Ann Surg. 2020;271(4):693‐700. 30308614 10.1097/SLA.0000000000002985

[20] Mirili C , Yilmaz A , Demirkan S , Bilici M , Basol TS . Clinical significance of prognostic nutritional index (PNI) in malignant melanoma. Int J Clin Oncol. 2019;24(10):1301‐1310. 31073814 10.1007/s10147-019-01461-7

[21] Wang D , Hu X , Xiao L , et al. Prognostic nutritional index and systemic immune‐inflammation index predict the prognosis of patients with HCC. J Gastrointest Surg. 2021;25(2):421‐427. 32026332 10.1007/s11605-019-04492-7 PMC7904713

[22] Wang Z , Wang Y , Zhang X , Zhang T . Pretreatment prognostic nutritional index as a prognostic factor in lung cancer: review and meta‐analysis. Clin Chim Acta. 2018;486:303‐310. 30138620 10.1016/j.cca.2018.08.030

[23] Cui J , Chen S , Bo Q , et al. Preoperative prognostic nutritional index and nomogram predicting recurrence‐free survival in patients with primary non‐muscle‐invasive bladder cancer without carcinoma in situ. Onco Targets Ther. 2017;10:5541‐5550. 29200869 10.2147/OTT.S146990 PMC5702160

[24] Li B , Lu Z , Wang S , et al. Pretreatment elevated prognostic nutritional index predicts a favorable prognosis in patients with prostate cancer. BMC Cancer. 2020;20(1):361. 32349713 10.1186/s12885-020-06879-1 PMC7191702

[25] Peng D , Gong YQ , Hao H , et al. Preoperative prognostic nutritional index is a significant predictor of survival with bladder cancer after radical cystectomy: a retrospective study. BMC Cancer. 2017;17(1):391. 28578683 10.1186/s12885-017-3372-8 PMC5455109

[26] Qi F , Zhou X , Wang Y , et al. Pre‐treatment prognostic nutritional index may serve as a potential biomarker in urinary cancers: a systematic review and meta‐analysis. Cancer Cell Int. 2018;18:207. 30564063 10.1186/s12935-018-0708-7 PMC6296044

[27] Yu J , Hong B , Park JY , Hwang JH , Kim YK . Impact of prognostic nutritional index on postoperative pulmonary complications in radical cystectomy: a propensity score‐matched analysis. Ann Surg Oncol. 2021;28(3):1859‐1869. 32776190 10.1245/s10434-020-08994-6 PMC7415333

[28] Bi H , Shang Z , Jia C , et al. Predictive values of preoperative prognostic nutritional index and systemic immune‐inflammation index for Long‐term survival in high‐risk non‐muscle‐invasive bladder cancer patients: a single‐Centre retrospective study. Cancer Manag Res. 2020;12:9471‐9483. 33061634 10.2147/CMAR.S259117 PMC7534864

[29] Templeton AJ , McNamara MG , Seruga B , et al. Prognostic role of neutrophil‐to‐lymphocyte ratio in solid tumors: a systematic review and meta‐analysis. J Natl Cancer Inst. 2014;106(6):dju124. 24875653 10.1093/jnci/dju124

[30] Viers BR , Boorjian SA , Frank I , et al. Pretreatment neutrophil‐to‐lymphocyte ratio is associated with advanced pathologic tumor stage and increased cancer‐specific mortality among patients with urothelial carcinoma of the bladder undergoing radical cystectomy. Eur Urol. 2014;66(6):1157‐1164. 24630414 10.1016/j.eururo.2014.02.042

[31] Buzby GP , Mullen JL , Matthews DC , Hobbs CL , Rosato EF . Prognostic nutritional index in gastrointestinal surgery. Am J Surg. 1980;139(1):160‐167. 7350839 10.1016/0002-9610(80)90246-9

[32] Munbauhal G , Drouin SJ , Mozer P , et al. Malnourishment in bladder cancer and the role of immunonutrition at the time of cystectomy: an overview for urologists. BJU Int. 2014;114(2):177‐184. 24410904 10.1111/bju.12529

[33] Tobert CM , Hamilton‐Reeves JM , Norian LA , et al. Emerging impact of malnutrition on surgical patients: literature review and potential implications for cystectomy in bladder cancer. J Urol. 2017;198(3):511‐519. 28286066 10.1016/j.juro.2017.01.087 PMC5705177

[34] Burden S , Billson HA , Lal S , Owen KA , Muneer A . Perioperative nutrition for the treatment of bladder cancer by radical cystectomy. Cochrane Database Syst Rev. 2019;5:CD010127. 31107970 10.1002/14651858.CD010127.pub2 PMC6527181

[35] Joseph N , Dovedi SJ , Thompson C , et al. Pre‐treatment lymphocytopaenia is an adverse prognostic biomarker in muscle‐invasive and advanced bladder cancer. Ann Oncol. 2016;27(2):294‐299. 26578732 10.1093/annonc/mdv546

[36] Petrie HT , Klassen LW , Kay HD . Inhibition of human cytotoxic T lymphocyte activity in vitro by autologous peripheral blood granulocytes. J Immunol. 1985;134(1):230‐234. 3871101

[37] el‐Hag A , Clark RA . Immunosuppression by activated human neutrophils. Dependence on the myeloperoxidase system. J Immunol. 1987;139(7):2406‐2413. 2821114

[38] McCourt M , Wang JH , Sookhai S , Redmond HP . Proinflammatory mediators stimulate neutrophil‐directed angiogenesis. Arch Surg. 1999;134(12):1325‐1331. discussion 31‐2. 10593330 10.1001/archsurg.134.12.1325

[39] Di Carlo E , Forni G , Musiani P . Neutrophils in the antitumoral immune response. Chem Immunol Allergy. 2003;83:182‐203. 12947985 10.1159/000071561

[40] McCourt M , Wang JH , Sookhai S , Redmond HP . Activated human neutrophils release hepatocyte growth factor/scatter factor. Eur J Surg Oncol. 2001;27(4):396‐403. 11417987 10.1053/ejso.2001.1133

[41] Jabłońska E , Kiluk M , Markiewicz W , Piotrowski L , Grabowska Z , Jabłoński J . TNF‐alpha, IL‐6 and their soluble receptor serum levels and secretion by neutrophils in cancer patients. Arch Immunol Ther Exp (Warsz). 2001;49(1):63‐69. 11266093

[42] Schaider H , Oka M , Bogenrieder T , et al. Differential response of primary and metastatic melanomas to neutrophils attracted by IL‐8. Int J Cancer. 2003;103(3):335‐343. 12471616 10.1002/ijc.10775

[43] Shamamian P , Schwartz JD , Pocock BJ , et al. Activation of progelatinase A (MMP‐2) by neutrophil elastase, cathepsin G, and proteinase‐3: a role for inflammatory cells in tumor invasion and angiogenesis. J Cell Physiol. 2001;189(2):197‐206. 11598905 10.1002/jcp.10014

[44] Scapini P , Nesi L , Morini M , et al. Generation of biologically active angiostatin kringle 1–3 by activated human neutrophils. J Immunol. 2002;168(11):5798‐5804. 12023382 10.4049/jimmunol.168.11.5798

[45] Lotan Y , Gupta A , Shariat SF , et al. Lymphovascular invasion is independently associated with overall survival, cause‐specific survival, and local and distant recurrence in patients with negative lymph nodes at radical cystectomy. J Clin Oncol. 2005;23(27):6533‐6539. 16116151 10.1200/JCO.2005.05.516

[46] Mari A , Kimura S , Foerster B , et al. A systematic review and meta‐analysis of lymphovascular invasion in patients treated with radical cystectomy for bladder cancer. Urol Oncol. 2018;36(6):293‐305. 29685374 10.1016/j.urolonc.2018.03.018

[47] Mertens LS , Claps F , Mayr R , et al. Prognostic markers in invasive bladder cancer: FGFR3 mutation status versus P53 and KI‐67 expression: a multi‐center, multi‐laboratory analysis in 1058 radical cystectomy patients. Urol Oncol. 2022;40(3):110.e1‐e9. 10.1016/j.urolonc.2021.10.010 34906411

[48] Mori K , Abufaraj M , Mostafaei H , et al. A systematic review and meta‐analysis of variant histology in urothelial carcinoma of the bladder treated with radical cystectomy. J Urol. 2020;204(6):1129‐1140. 32716694 10.1097/JU.0000000000001305

[49] Claps F , van de Kamp MW , Mayr R , et al. Prognostic impact of variant histologies in urothelial bladder cancer treated with radical cystectomy. BJU Int. 2023. 10.1111/bju.15984 36748180

[50] Moschini M , Dell'Oglio P , Luciano R , et al. Incidence and effect of variant histology on oncological outcomes in patients with bladder cancer treated with radical cystectomy. Urol Oncol. 2017;35(6):335‐341. 28087131 10.1016/j.urolonc.2016.12.006

[51] Claps F , van de Kamp MW , Mayr R , et al. Risk factors associated with positive surgical margins' location at radical cystectomy and their impact on bladder cancer survival. World J Urol. 2021;39(12):4363‐4371. 34196758 10.1007/s00345-021-03776-5

[52] Marcq G , Afferi L , Neuzillet Y , et al. Oncological outcomes for patients harboring positive surgical margins following radical cystectomy for muscle‐invasive bladder cancer: a retrospective multicentric study on behalf of the YAU urothelial group. Cancers (Basel). 2022;14(23):5740. 36497222 10.3390/cancers14235740 PMC9739538

